# Editorial: Cellular stress in blood cancer: Pathophysiology and therapeutic opportunities

**DOI:** 10.3389/fonc.2022.1026315

**Published:** 2022-09-29

**Authors:** José A. Costoya, Francesco Piazza

**Affiliations:** ^1^ Molecular Oncology Laboratory MOL, Departamento de Fisioloxía, Centro Singular de Investigación en Medicina Molecular e Enfermidades Crónicas (CiMUS), Facultade de Medicina, Universidade de Santiago de Compostela, Instituto de Investigación Sanitaria de Santiago de Compostela (IDIS), Santiago de Compostela, Spain; ^2^ Laboratory of Myeloma and Lymphoma Pathobiology, Unit of Hematological Malignancies, Veneto Institute of Molecular Medicine – Foundation for Advanced Biomedical Research, Padua, Italy; ^3^ Division of Hematology, Department of Medicine, Azienda Ospedale Università di Padova, University of Padua, Padua, Italy

**Keywords:** blood malignancies, cellular stress, DNA damage response, reactive oxygen species, leukemogenesis, therapeutic targets

The cell can mitigate the possible damage induced by oxidative and other toxic agents preserving its survival. The activation of different pathways, with the purpose of maintaining key cellular functions, will assure the cellular homeostasis despite a hostile environment. Among the intracellular mechanisms that are activated in response to different stresses are DNA damage response (DDR), mitochondrial stress signaling and autophagy, and in many cases these mechanisms will regulate cell cycle, cell death response and senescence that elicit a systemic response (1). These responses produce modifications of stressed cells associated to secretion of soluble factors or microvesicles, that in some cases are linked to disease, such as cancer (1). In fact, cancer cells exploit these responses to adapt and survive to many types of dangerous *noxae* related to the oncogenic process, such as hypoxia, nutrient deprivation, and the associated metabolic and oxidative stress. These mechanisms are key for cell survival allowing cancer progression and resistance to therapy, as well as evasion of immune surveillance. Thus, the understanding of the molecular mechanisms of cancer response to stress can provide fundamental insights to recognize new potential therapeutic targets and improve clinical management (2).

The Research Topic *Cellular Stress in Blood Cancer: Pathophysiology and Therapeutic Opportunities* has collected original contributions that deal with newest pathogenetic aspects of blood cancer. In the era of molecular genomics that has changed the taxonomy of blood tumors, it has become important to exploit the large amount of new information to scrutinize relevant pathobiological mechanisms amenable of therapeutic targeting. Indeed, it has become clear that both chronic and acute leukemias are characterized by tumor-intrinsic and cell-extrinsic, microenvironmental alterations. Capturing the complexity of such changes and dissecting pathogenic clues is the aim of current research in the field. The contributions to the present Research Topic are clearly devoted to this purpose (**Figure 1**).

**Figure 1:**
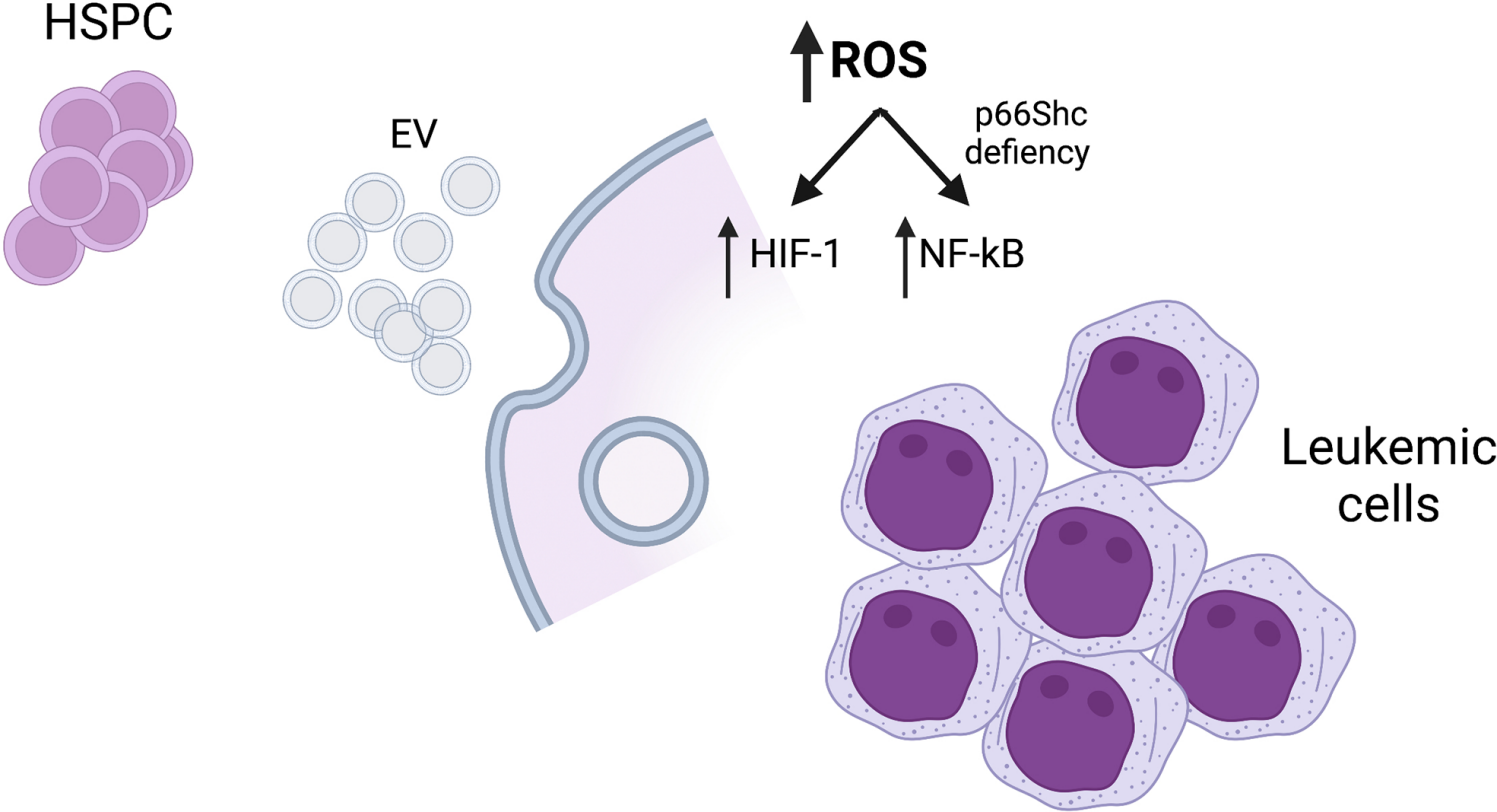
Mechanisms of cellular stress responses in blood malignancies. HSPC, hematopoietic stem/progenitor cells; EV, extracellular vesicles; ROS, reactive oxygen species. Created with BioRender.com.

In the paper by Trino et al., intriguing findings establish a pathophysiological mechanisms whereby acute myeloid leukemia cells may affect normal hematopoiesis. It was known that acute myeloid leukemia (AML) is characterized by a compromise of the differentiation of the remaining normal bone marrow hematopoietic cells and several mechanisms have been invoked to explain this phenomenon (3). Aside the case in which bone marrow is substituted by AML blasts so that no space is left for normal hematopoietic cells, other mechanisms are involved in those AML cases without massive bone marrow infiltration. Trino et al. provide evidence that AML blasts modify the gene expression profile through the secretion of extracellular vesicles (EV) in which are contained miRNAs with relevant pathogenic roles. Hematopoietic stem/progenitor cells (HSPC) thus receive aberrant signals brought by miRNA-associated and other components in EV that induce profound phenotypic changes (both at the level of critical transcription factors, signaling pathways, metabolism and surface molecules) and substantial skewing in their differentiation potential (with accumulation of earlier precursors in spite of more differentiated ones). Moreover, modified HSPC secrete cytokines such as IL-1, CCL3 and GM-CSF that cause an inflammatory microenvironment.

The concept of EVs has vastly changed during the last years. Currently, these lipid bilayer particles are considered highly heterogeneous, not only in terms of biogenesis but also in size and content. These vesicles are crucial for cell-to-cell communication of survival signals in response to stress signals and resistance to therapy (4). Thus, this new mechanism would be amenable of potential novel therapeutic approaches

Reactive oxygen species (ROS), generated during cellular response to oxidative stress, modulate the function of many transcription factors. Among these, NF-κB signaling is regulated by the levels of these species, activating or inhibiting its function. The complexity of ROS-dependent pathways and the cellular context determine the final output. Thus, ROS levels must be precisely regulated both in normal and leukemic cells (5, 6). In the paper by Tatangelo et al., the subject of the regulation of intracellular oxidative stress and apoptosis by the protein p66Shc has been investigated in chronic lymphocytic leukemia (CLL), the most frequent type of leukemia in adults in Western countries (7). The paper clearly dissects a pathway ending up in the activation of the transcription factor NF-kB and transcriptional upregulation of critical chemokine receptors CCR2, CXCR3 and CCR7, which are exploited by leukemic cells to recirculate in the lymphatic tissue and escape pro-death microenvironment. Mechanistically, it is demonstrated that p66Shc might repress NF-kB activity through the generation of ROS. Loss of p66Shc, which is frequently observed in CCL cells, is therefore associated to an enhanced NF-kB activity and survival of CLL cells.

This distorted response produced by the p66Shc deficiency can be reversed by the p66Shc reconstitution in CLL cells, normalizing the NF-kB activation by ROS in CLL cells. The article highlights the pathogenic role of the alterations of stress signaling pathways in blood tumors.

The two other contributions deal with the cellular response to hypoxia, mainly based on Hypoxia-inducible factors (HIFs), and the role of these transcription factors in hematologic malignancies. The transcription factor HIF-1 is composed by an HIF-1α subunit, which binds the HIF-1β subunit and drives the transcription of genes involved in metabolism and angiogenesis, increasing the oxygen supply through the stabilization of HIF-1α in the presence of low oxygen levels. Previous studies have demonstrated that abnormal production of ROS in cancer cells is associated to the stabilization of HIF-1α, promoting tumor progression and invasion (8). In chronic myeloid leukemia (CML) cell populations, HIF-1α and HIF-responsive genes are upregulated by the oncoprotein BCR/-ABL1. Moreover, the transcription factor HIF1α appears as a promising target for the management of many cancers. In the paper by Singh et al., the authors have investigated the intricate network that links HIF-1α to several stress and signaling pathways in CML. The authors demonstrate an ongoing balance of oxidant and antioxidant factors, an upregulation of metabolic pathways which stabilize HIF1a that in turn drives the transcription of a plethora of important target genes. Moreover, an intriguing link of HIF-1α to the Notch pathway, and in particular Notch1, with important pathogenetic and therapeutic consequences in CML is established.

In the comprehensive review by Magliulo and Bernardi the knowledge on the roles of HIFs in cell physiology and in the pathogenesis of blood tumors is exhaustively described. The authors highlight the fact that in hematological malignancies as opposed to solid tumors the field is in its dawn, yet it bears important implications in the pathophysiology of such malignancies as well as provides clear facets of potential therapeutic applications. The review describes HIFs in acute and chronic myeloid and lymphoid malignancies.

Altogether, the papers in this Research Topic indicate that stress pathways in blood tumors are involved in many pathogenic aspects and are amenable of therapeutic intervention. Future studies will further clarify the interconnections of stress pathways with the different genetic subtypes of the several blood cancers described so far are needed to identify discrete pathways on which it would be possible to intervene with novel treatments.

## Author contributions

All authors listed have equally contributed and approved it for publication.

## Funding

This work received financial support from the Ministerio de Ciencia e Innovación (PID2020-113501RB-I00; JAC), the Centro Singular de Investigación de Galicia accreditation 2016–2019, ED431G/05) and the European Regional Development Fund (ERDF). FP is supported by grants from Associazione Italiana per la Ricerca sul Cancro (AIRC) and Ministry of Research (Progetti di RIlevante Interesse Nazionale, PRIN).

## Conflict of interest

The authors declare that the research was conducted in the absence of any commercial or financial relationships that could be construed as a potential conflict of interest.

## Publisher’s note

All claims expressed in this article are solely those of the authors and do not necessarily represent those of their affiliated organizations, or those of the publisher, the editors and the reviewers. Any product that may be evaluated in this article, or claim that may be made by its manufacturer, is not guaranteed or endorsed by the publisher.
